# Engaging Operational Partners Is Critical for Successful Implementation of Research Products: a Coincidence Analysis of Access-Related Projects in the Veterans Affairs Healthcare System

**DOI:** 10.1007/s11606-023-08115-5

**Published:** 2023-06-20

**Authors:** Jessica R. Dodge, Bradley Youles, Jennifer Caldararo, Erika D. Sears, Tanner J. Caverly, P. Michael Ho, Stephanie L. Shimada, Peter Kaboli, Karen Albright, Stephanie A. Robinson, Demetria M. McNeal, Laura Damschroder, Sameer D. Saini, Megan A. Adams

**Affiliations:** 1 Center for Clinical Management Research, VA Ann Arbor Healthcare System, Ann Arbor, MI USA; 2 University of Michigan Medical School, Ann Arbor, MI USA; 3 Institute for Healthcare Policy and Innovation, Ann Arbor, MI USA; 4 Denver-Seattle Center of Innovation for Veteran-Centered and Value-Driven Care, Rocky Mountain Regional VA Medical Center, Aurora, CO USA; 5 VA Center for Healthcare Organization and Implementation Research, VA Bedford Healthcare System, Bedford, MA USA; 6 Iowa City VA Center for Access and Delivery Research and Evaluation (CADRE), Iowa VA Healthcare System, Iowa City, IA USA; 7 Division of General Internal Medicine, University of Colorado School of Medicine, Aurora, CO USA; 8 The Pulmonary Center, Boston University School of Medicine, Boston, MA USA; 9 Department of Internal Medicine, Division of Gastroenterology, University of Michigan, Ann Arbor, MI USA

**Keywords:** Access, Implementation, Veterans

## Abstract

**Background/Objective:**

The Veterans Health Administration (VHA) has prioritized timely access to care and has invested substantially in research aimed at optimizing veteran access. However, implementing research into practice remains challenging. Here, we assessed the implementation status of recent VHA access-related research projects and explored factors associated with successful implementation.

**Design:**

We conducted a portfolio review of recent VHA-funded or supported projects (1/2015–7/2020) focused on healthcare access (“Access Portfolio”). We then identified projects with implementable research deliverables by excluding those that (1) were non-research/operational projects; (2) were only recently completed (i.e., completed on or after 1/1/2020, meaning that they were unlikely to have had time to be implemented); and (3) did not propose an implementable deliverable. An electronic survey assessed each project’s implementation status and elicited barriers/facilitators to implementing deliverables. Results were analyzed using novel Coincidence Analysis (CNA) methods.

**Participants/Key Results:**

Among 286 Access Portfolio projects, 36 projects led by 32 investigators across 20 VHA facilities were included. Twenty-nine respondents completed the survey for 32 projects (response rate = 88.9%). Twenty-eight percent of projects reported fully implementing project deliverables, 34% reported partially implementing deliverables, and 37% reported not implementing any deliverables (i.e., resulting tool/intervention not implemented into practice). Of 14 possible barriers/facilitators assessed in the survey, two were identified through CNA as “difference-makers” to partial or full implementation of project deliverables: (1) engagement with national VHA operational leadership; (2) support and commitment from local site operational leadership.

**Conclusions:**

These findings empirically highlight the importance of operational leadership engagement for successful implementation of research deliverables. Efforts to strengthen communication and engagement between the research community and VHA local/national operational leaders should be expanded to ensure VHA’s investment in research leads to meaningful improvements in veterans’ care.

**Lay Summary:**

The Veterans Health Administration (VHA) has prioritized timely access to care and has invested substantially in research aimed at optimizing veteran access. However, implementing research findings into clinical practice remains challenging, both within and outside VHA. Here, we assessed the implementation status of recent VHA access-related research projects and explored factors associated with successful implementation. Only two factors were identified as “difference-makers” to adoption of project findings into practice: (1) engagement with national VHA leadership or (2) support and commitment from local site leadership. These findings highlight the importance of leadership engagement for successful implementation of research findings. Efforts to strengthen communication and engagement between the research community and VHA local/national leaders should be expanded to ensure VHA’s investment in research leads to meaningful improvements in veterans’ care.

**Supplementary Information:**

The online version contains supplementary material available at 10.1007/s11606-023-08115-5.

## INTRODUCTION

Ensuring timely, high-quality access to care for US military veterans has been a longstanding priority of the Veterans Health Administration (VHA), and continues to be a prominent focus of the VHA Strategic Plan.^1^ VHA has invested substantially in access-related research and operational initiatives over many years to further this goal.^2–5^ Recent legislative efforts to expand the eligibility of VHA-enrolled veterans for community care, including the Veterans Access, Choice, and Accountability Act of 2014 (“Choice Act”)^6^ and VA Maintaining Internal Systems and Strengthening Integrated Outside Networks (MISSION) Act of 2018,^7^ also are designed to expand veterans’ options for accessing needed healthcare services in non-VHA facilities. Likewise, VHA’s substantial investment in telehealth, which preceded the COVID-19 pandemic but has expanded in its wake, also aimed to facilitate improvements in access.^8^ Despite some evidence of progress, VHA faces ongoing challenges in optimizing veteran access to care exacerbated by the COVID-19 pandemic and related disruptions to the global healthcare system.^9,10^

In 2020, VHA Health Services Research & Development (HSR&D) funded the Veterans Access Research Consortium (VARC) to accelerate translation of access-related research to clinical care settings and stimulate measurable improvements in access to care.^11^ A core goal of VARC was to undertake a portfolio review of existing VHA access-related projects to understand the current state of access-related initiatives in VHA and their implementation stage. In this study, we sought to build upon VARC’s portfolio review by employing novel implementation science methods to elucidate factors or combinations of factors critical to a successful implementation of access-related research projects in VHA.^12,13^

## METHODS

The project proceeded in 4 steps: (1) a portfolio review of recent VHA projects focused on improving veteran access to care; (2) identification of a subset of projects meeting specific study inclusion criteria; (3) development and administration of an electronic survey to assess each project’s implementation status and identify barriers and facilitators to implementation of deliverables (defined as effective interventions or usable tools that constitute the main work product of the project); and (4) analysis of survey data to empirically identify key factors or combinations of factors associated with successful implementation, as detailed below.

### Portfolio Assessment

A portfolio review of recent VHA projects focused on access to care (“Access Portfolio”) was conducted. Specifically, we systematically searched VHA and National Library of Medicine websites and conducted structured interviews with VHA operational partners and researchers to identify all projects funded or supported by VHA (e.g., Health Services Research & Development (HSR&D) (investigator-initiated research), Quality Enhancement Research Initiative (QUERI) (partnered research), or funded by a national program office (non-research)) between 1/2015 and 7/2020 that were directly or indirectly related to access to care. We chose a start date of 1/2015 to capture projects funded following increased scrutiny of the VHA over timely access to care relating to the 2014 VHA wait-time scandal.^14^ We then identified projects with implementable research deliverables by excluding those that (1) were non-research/operational projects; (2) were only recently completed (i.e., completed on or after 1/1/2020, meaning that they were unlikely to have had time to be implemented); and (3) did not propose an implementable deliverable (e.g., observational research that did not result in a specific intervention or usable tool). We excluded purely operationally funded projects because these projects, by nature of being operationally funded/operationally driven, presumably had strong operational support, as compared to investigator-initiated projects. Partnered research (e.g., program evaluations) funded through the VA Quality Enhancement Research Initiative were included because they were not purely operationally funded.

The study team developed and refined a rubric to categorize each project in the portfolio (Appendix 1). Projects were classified by project characteristics including study design (observational, program evaluation, interventional), complexity of the primary site where the project was conducted using the VHA facility complexity score,^15^ and type of clinical care setting (i.e., primary care, specialty care, mental health care, inpatient/acute care, other). Projects also were classified based on elements specific to access, including whether they were “access-specific” (i.e., both relevant to access and incorporated specific measure(s) of access) or “access-relevant” (i.e., relevant to access, but did not specifically assess/measure access), and the dimension (domain) of access on which they focused (if any). Regarding access domains, we incorporated into our classification rubric the 5 domains of access included in the Fortney model, including geographical (ease of traveling to healthcare provider locations), temporal (time required to receive services and the opportunity cost of that time), financial (healthcare system eligibility issues and the cost of utilizing healthcare services), cultural (acceptability of healthcare services), and digital (connectivity that enables synchronous or asynchronous digital communications with formal providers, informal caregivers, peers, and computerized health applications).^16^ The Fortney model is a well-recognized framework that conceptualizes access to care as the fit between the individual and the healthcare system and highlights the importance of understanding and measuring both actual and perceived access.^16^ Projects also were classified by the operational priority area(s) they addressed, if any, and by whether they involved VHA community care or virtual care.

Two study team members (BY, JC) independently reviewed and coded each project included in the portfolio assessment. Discrepancies were initially resolved by consensus among the two primary reviewers. To ensure fidelity in project classification, 20% of all projects were reviewed by a subset of investigators (MA, ES, TC, SS), with a specific focus on those where a discrepancy in project classification existed among the two primary reviewers (i.e., all projects where a discrepancy between the two primary reviewers existed were reviewed by the four investigators).

### Implementation Survey

After the portfolio review process, a 24-item electronic survey (Appendix 2) was developed by the study team and administered to the designated principal investigators and/or project leads for each project identified during the portfolio review using Qualtrics software.^17^ The survey was designed to assess the implementation status of each access portfolio project meeting study criteria (“Has the deliverable from your project been implemented either in whole or in part?”), and to identify specific barriers and facilitators to the implementation of project deliverables. In this context, we specifically defined project deliverables to be effective interventions or usable tools that constituted the central work product of the project. The survey included both closed and open-ended questions. Survey development was informed by the Consolidated Framework for Implementation Research (CFIR), which provides a menu of constructs across five domains (innovation, inner setting, individuals, outer setting, process) associated with effective implementation.^18,19^ The CFIR was developed as a theory-based way to understand the context within which evidence-based programs are implemented.^18,19^

A draft survey instrument was developed and refined by the project team before finalization and dissemination. Principal investigators (PI) and/or project leads identified in the portfolio review were invited to participate between April 2021 and June 2021. Only projects that ended before 2020 were assessed via the survey to ensure that all projects were afforded sufficient time to implement deliverables (i.e., at least a year after completion of the funding period/project term).

### Analysis of Survey Results

Python packages Pandas (version 1.3.2) and NumPy (version 1.21.2) were used to analyze survey data and report descriptive statistics. To empirically identify factors or combinations of factors critical for implementation success, we analyzed survey data using the novel Coincidence Analysis (CNA) methodology.^20^ CNA is a new but increasingly established method in implementation science research.^12,13,21–23^ CNA is a configurational approach to analysis, meaning that it does not rely on incremental differences between an independent (X) and dependent variable (Y) as in correlational approaches.^24^ Instead, CNA is a set-theoretical analytic approach that uses Boolean algebra to evaluate how *combinations* of factors (known in CNA as conditions) may lead to an outcome of interest. CNA searches for causal relations between conditions and the outcome of interest to find “difference-maker” conditions or combinations of conditions that lead to the outcome.^12,24^ In this case, the outcome of interest was a full or partial implementation of an access-related deliverable.^25^ CNA identifies how multiple conditions work together in configurations that operate jointly and allow for modeling multiple paths leading to an outcome (equifinality) as well as when a condition may only be relevant to an outcome if it is paired with another condition (causal complexity).^25,26^ The ability of CNA to detect equifinality and complex causality makes it ideal to assess facilitators and barriers to implementation success. CNA is a unique Configurational Comparative Methodology that uses a bottom-up algorithm designed for application in social science research.^24^ Furthermore, CNA has the ability for factor selection also making it ideal when there are a variety of potentially relevant factors to the outcome. R Studio, R, and the “cna” package were used for CNA, and the “msc” function was used for factor selection.

This study was considered non-research quality improvement based on VHA policy designating non-research projects for VHA system improvement. As such, it was exempt from Institutional Review Board review.

## RESULTS

In the larger portfolio assessment, the two primary reviewers agreed on project classification in 88% of cases, with all cases where disagreement existed resolved by consensus as outlined above.

Of the 286 projects in the Access Portfolio, 250 were excluded because they had a completion date on or after 1/1/2020 (*n* = 186), were purely operationally funded (*n* = 60), and/or did not propose a specific project deliverable (*n* = 35). Thirty-six projects led by 32 unique investigators across 20 VHA facilities were included in the survey. Twenty-nine respondents (PIs/project leads) completed the survey (response rate 88.9%) for 32 projects. Summary characteristics of these projects are presented in Table 1. (Project-specific details are included in Appendix 3.)

**Table 1:** Access Project Characteristics

Characteristic			N (%)/M (SD)
Site demographics	Facility complexity level	1a	24 (75%)
		1b	5 (16%)
		1c	1 (3%)
		3	2 (6%)
Project characteristics	Clinical care setting	Primary care	7 (22%)
		Specialty care	7 (22%)
		Mental health	10 (31%)
		Inpatient/acute care	1 (3%)
		Other care not listed	7 (22%)
	Study design^+^	Observational	5 (16%)
		Program evaluation	10 (31%)
		Interventional	17 (53%)
	Reported implementation status (survey)	Completely implemented	11 (34%)
		Partially implemented	12 (37%)
		Not implemented	9 (28%)
Elements specific to access	Fortney access model domain (only access-specific projects classified; projects not limited to single domain)	Geographical	6 (19%)
		Temporal	2 (6%)
		Cultural	2 (6%)
		Digital	5 (16%)
		Financial	3 (9%)
	Degree of focus on access	Access-relevant	24 (75%)
		Access-specific:	8 (25%)
		Actual access	4 (50%)
		Perceived access	4 (50%)
	Operational priority area (top 2 priority areas, where project relevant to multiple priority areas)	System redesign (e.g., Patient Aligned Care Team integration, clinical delegation, MISSION)	7 (22%)
		Overuse/low-value care/appropriateness	1 (3%)
		Prioritization/urgency/waitlist management	1 (3%)
		Virtual care/technology	20 (63%)
		Burnout	0 (0%)
		Workforce satisfaction/retention/expansion	5 (16%)
		Clinical operations	6 (19%)
		Access measurement	3 (9%)
		Improving patient satisfaction/experience (must have product to address satisfaction)	6 (19%)
		None (did not map to any specified operational priority area)	2 (6%)
	Focus VHA community care or virtual care	MISSION Act	0 (0%)
		Veterans’ choice act/program	1 (3%)
		Other non-VHA data or care (e.g., Medicare, civilian care WITHOUT a project focus on MISSION or choice)	2 (6%)
		Virtual care (e.g., telehealth, MyHealtheVet, apps, etc.)	20 (63%)
		None	9 (28%)

^+^Study design definitions are as follows: observational—secondary data analysis, mixed methods, qualitative methods, or modeling; program evaluation—evaluation of a programmatic initiative designed to improve access; interventional—prospective evaluation of an intervention designed to improve access. These definitions are outlined further in the following publication: Peters, W. S. Observational Studies and Program Evaluation. In: Counting for Something. Springer Texts in Statistics. 1987. Springer, New York, NY

In terms of study design, most projects were interventional (17/32; 53%), and the remainder were program evaluations (10/32; 31%) or observational (5/32; 16%). The majority were related to mental health care (10/32; 31%), followed by primary care and specialty care (22% of projects each), and were focused on virtual care/technology (i.e., 63% of projects had either a primary or secondary theme of virtual care/technology, a priority area of the former VHA Office of Veterans Access to Care). Most projects were access-relevant (24/32; 75%) rather than access-specific. In terms of implementation status, 34% of projects (11/32) reported having fully implemented project deliverables, 37% of projects (12/32) reported partially implementing project deliverables, and 28% of projects (9/32) reported not implementing any deliverables (i.e., the resulting tool/intervention was not implemented into practice).

Table 2 presents responses to survey questions mapped to CFIR domains. Most PIs/project leads identified the following as facilitators of implementation: having sufficient resources (70%), information technology (IT) support (62%), local site operational leadership support and commitment (63%), national VHA operational partner support and commitment (70%), presence of a local “champion” (61%), and contacting or sharing deliverables with national VHA operational offices (i.e., “engagement” with national VHA operational leadership) (66%). Reported barriers to implementation included limitations on the PI/project lead’s time, expertise, or resources (52%), changes in the environment (70%), and not contacting/engaging operational leadership at the local level (87%) or regional level (91%).

**Table 2:** Survey Question Responses Mapped to Consolidated Framework for Implementation Research (CFIR) Constructs

CFIR 2.0domain	Question		N (%)/M (SD)
Innovation	The deliverable has required substantial modifications prior to broader implementation	Strongly or somewhat disagree	9 (43%)
		Neither agree nor disagree	3 (14%)
		Strongly or somewhat agree	9 (43%)
Outer setting & inner setting	Implementation has had sufficient resources (e.g., equipment, staff)	Strongly or somewhat disagree	3 (13%)
		Neither agree nor disagree	4 (17%)
		Strongly or somewhat agree	16 (70%)
	Implementation has had sufficient IT support	Strongly or somewhat disagree	4 (19%)
		Neither agree nor disagree	4 (19%)
		Strongly or somewhat agree	13 (62%)
	Implementation would create tension with existing practice (e.g., standards of care, guidelines, policies, systems)	Strongly or somewhat disagree	14 (54%)
		Neither agree nor disagree	3 (12%)
		Strongly or somewhat agree	9 (35%)
	Changes in environment have rendered implementation less relevant	Strongly or somewhat disagree	19 (70%)
		Neither agree nor disagree	4 (15%)
		Strongly or somewhat agree	4 (15%)
	Implementation has had support and buy-in from key outside community entities	Strongly or somewhat disagree	5 (29%)
		Neither agree nor disagree	8 (47%)
		Strongly or somewhat agree	4 (24%)
	Operational offices either contacted or deliverable shared (“engagement”)	Local/medical center	4 (13%)
		Regional/visn	3 (9%)
		National/VHA	21 (66%)
Individuals (outer setting)	Implementation has had support and commitment from national VHA operational leadership	Strongly or somewhat disagree	3 (13%)
		Neither agree nor disagree	4 (17%)
		Strongly or somewhat agree	16 (70%)
Individuals (inner setting)	Implementation has been limited by PI’s time,expertise,and**/**or resources	Strongly or somewhat disagree	13 (52%)
		Neither agree nor disagree	3 (12%)
		Strongly or somewhat agree	9 (36%)
	Implementation has had buy-in from frontline providers and staff	Strongly or somewhat disagree	5 (21%)
		Neither agree nor disagree	7 (29%)
		Strongly or somewhat agree	12 (50%)
	Implementation has had support and commitment from local site operational leadership	Strongly or somewhat disagree	3 (13%)
		Neither agree nor disagree	6 (25%)
		Strongly or somewhat agree	15 (63%)
	Implementation has had a champion at the implementation site	Strongly or somewhat disagree	4 (17%)
		Neither agree nor disagree	5 (22%)
		Strongly or somewhat agree	14 (61%)

Out of 14 possible barriers and facilitators (Table 2), factor selection revealed nine suitable for full CNA analysis. CNA revealed only two to be “difference-makers” that led to partial or full implementation of project deliverables: (1) engagement with national VHA operational leadership or (2) support and commitment from local site operational leadership. These two environmental conditions (not collectively, but independently) explained 91% of projects with full or partial implementation with 100% accuracy. In other words, when all potential factors were assessed configurationally through Boolean algebra in relation to the outcome, engagement with national VHA operational leadership or support and commitment from local site operational leadership were present in 91% of the projects assessed, and 100% of those projects achieved partial or full implementation.

## DISCUSSION

In this study, we leveraged a novel analytic method in implementation science, Coincidence Analysis, to better understand elements critical to the successful implementation of access-related research deliverables. Of all factors examined, only two—engagement with national VHA operational leadership or support and commitment from local site operational leadership (not both, but either)—were found to be “difference-makers” that led to full/partial implementation of access-related deliverables, in whole or in part, into clinical practice. Thus, our findings build off prior qualitative data and empirically demonstrate using novel quantitative methods the importance of close engagement and bi-directional collaboration with either national or local operational partners to successfully implement access-related research findings to improve care for veterans.

The importance of research-operational partnerships in facilitating the successful translation of patient-oriented research findings to practice has long been recognized. In a 2014 commentary, the director of VHA’s Quality Enhancement Research Initiative (QUERI) urged health services investigators to “partner or perish” and called on the research community to “actively promote alliances with program partners, and to ensure that frontline providers are actively involved in the development and implementation of new research initiatives to ensure uptake and impact.”^27^ Such engagement not only serves to increase the policy-relevance of research questions, but also fosters subsequent integration of findings into policy and practice.^28^

Operational partners are critical to successful implementation for several reasons. First, operational leaders are well-positioned to influence healthcare innovations given their role in developing and overseeing programs and policy directives, whether locally or nationally.^29^ Second, operational leaders possess decision-making authority on resource and staffing allocations critical to the successful implementation, scale-up, and sustainability of research deliverables.^29^ Engagement of operations leaders in research and other projects also is critical to ensure that research is designed and conducted to maximize alignment with operational needs and objectives. This is particularly true in access-related research, given the high-priority nature of access-related initiatives in furtherance of the VHA Strategic Plan.^1^

While the exact operational partners to be engaged (e.g., local operational leaders such as clinic directors and site-level service chiefs, or national operational leaders such as specialty program directors, policy office leadership, and others) will depend on the individual project’s aims and outcomes,^30^ our findings suggest that such partnerships are critical to successful translation of access-related initiatives into practice. Importantly, while many efforts to develop research-operational partnerships focus primarily on engaging national leaders, our findings demonstrate that investment in partnerships with local (site) leadership is just as important. Additional initiatives to promote research-operational partnerships at the local level, and to re-align incentives to support such engagement, could facilitate more effective dissemination and implementation of access-related research to enhance frontline veteran care. For example, developing a specific pathway to access supplemental funding within 2 years of the conclusion of the grant funding period tied to an implementation plan written with local, regional, or national operational leadership could serve as a powerful stimulus to aid the translation of research findings into clinical practice, and help counter situations where project investigators no longer have the funding to support ongoing implementation efforts.

Several recent studies have employed qualitative methods to elucidate essential components of successful research-operations partnerships in VHA, and our study builds upon these findings using novel quantitative methodology. In a recent qualitative study of investigators and operational partners involved in VHA QUERI National Partnered Evaluation Initiative projects, partnership characteristics found to facilitate effective collaboration included leadership support, shared understanding of planned work, investment, trust, and agreement on project deliverables.^30^ Likewise, key partnership strategies noted to be instrumental to the success of other QUERI-funded evidence-based practice implementation projects include (1) understanding different time horizons for addressing important clinical problems from research and policy perspectives; (2) identifying research questions that remain relevant to partners over time, (3) designing studies that are flexible as clinical systems change; (4) engagement of partners throughout the course of research; and (5) building relationships of mutual respect, trust, and credibility.^31^ While a specific framework to guide research-operation collaborations in VHA has yet to be developed, the adoption of a partnership model informed by empirically derived models for developing scalable interventions, such as the World Health Organization’s (WHO) ExpandNet framework, has been proposed.^29^ For example, the WHO ExpandNet framework was used to maximize the impact of research-operational partnerships in scaling up a brief Cognitive Behavioral Therapy intervention in VHA primary care clinics.^29^ Such a model also could aid the implementation of access-related initiatives in the future.

Our study has several limitations. First, our results may not generalize to non-VHA healthcare systems. However, the importance of strong partnerships between researchers and knowledge users in efforts to scale up interventions is well-recognized both within and outside VHA.^28,29^ Second, our results may have been confounded by unmeasured factors located on the causal pathway to implementation that do not go through any measured factors. These unmeasured factors may not affect all cases/configurations equally.^12^ In this case, there may be a risk of over-interpreting the data or incorrectly inferring a causal relationship where none exists. However, as outlined above, these results are logical given the role of operational stakeholders in allocating resources, defining priorities, and implementing policy initiatives. To confirm these findings, the relationship between leadership engagement and partial/full implementation can be further explored through qualitative interviews and other methods in the future. Second, our CNA analysis may have only revealed portions of the underlying causal structures. As such, just because specific factors (e.g., IT support, presence of a local “champion”) were not identified as relevant in CNA analysis of this data does not mean that these factors are causally irrelevant—configurational data analyzed in observational studies tend to exhibit low diversity such that most logically possible combinations of factors are not present in the observed cases.^12^ Lastly, all interventions are not created equal (i.e., they vary in their mechanism, scope, and impact), such that it is challenging to compare the impact of different interventions in terms of their degree of implementation (partial or full). However, our study demonstrates that, regardless of intervention type/characteristic, the same two difference-making factors support successful implementation.

## CONCLUSION

These findings suggest how critical an engagement strategy is for the successful implementation of research deliverables. Support and commitment from *either* national or local/site operational leadership are vital to successful implementation. Future work should explore ways to strengthen communication between the research community and VHA leaders, at multiple levels, to ensure that VHA’s investment in access-related projects leads to meaningful improvements in care delivery for veterans.

## Supplementary Information

Below is the link to the electronic supplementary material.

Supplementary file1 (DOCX 17 kb)

Supplementary file2 (DOCX 32 kb)

Supplementary file3 (DOCX 33 kb)

## Data Availability

Jennifer Burns at jennifer. burns@va. gov. They should state their reason for requesting the data and their plans for analysing the data. Final data sets will be copied onto a DVD. The DVD will be sent to the requester via FedEx. Each data set will be accompanied by documentation that lists all variables described in the publication and links them with variable names in the data set. De-identified data will be provided after requesters sign a letter of agreement (LOA) detailing the mechanisms by which the data will be kept secure. The LOA will also state that the recipient will not attempt to identify any individual in the data, will not share the data outside of their research team, and will provide information on any files to be linked to the data.
