# Supplementary material for: Engaging Operational Partners Is Critical for Successful Implementation of Research Products: a Coincidence Analysis of Access-Related Projects in the Veterans Affairs Healthcare System
Source: J Gen Intern Med. 2023 Jun 20;38(Suppl 3):923–30. doi: 10.1007/s11606-023-08115-5 (PMC10356702; doi:10.1007/s11606-023-08115-5)
Supplement: Supplementary file 1 — Supplementary file1 (DOCX 17 kb) [file 11606_2023_8115_MOESM1_ESM.docx]

**Appendix 1. Rubric Used by the Study Team to Classify Access Portfolio Projects**

| **Code 1: Care Setting (select one)** | | | |
| --- | --- | --- | --- |
| Type | | | Definition |
| Primary Care | | | The project is focused on primary care access |
| Specialty Care | | | The project is focused on specialty care access |
| Mental Health | | | The project is focused on mental health access |
| Inpatient Care | | | The project is focused on inpatient care |
| Long-term care | | | The project is focused on long-term care |
| Other care | | | The care setting focus consists of more than one type or is not described in the abstract. |
| **Code 2: Access specific versus Access relevant (select one)** | | | |
| Type | | | Definition |
| Access Specific | | | A project that has an access impact and measures actual or perceived access |
| Access Relevant | | | A project that has an access impact, but does not incorporate specific measurements of access |
| **Code 3: Access Distinction (select one)** | | | |
| Access Distinction | | | Definition |
| Actual Access | | | Direct measurement of access, usually measured admin data that are not self-reported (i.e., EHR, GIS, etc.) |
| Perceived Access | | | A measurement of perceived access, typically from a self-assessment (e.g., SHEP, V-Signals) |
| **Code 4: Tags (select all that apply) -> For Access-specific projects only** | | | |
| Tag(s) | | | Definition |
| Geographical | | | The primary purpose of the project is to address the difficulties of traveling to healthcare provider locations. |
| Temporal | | | Wait time due to appointment availability. |
| Cultural | | | The project evaluates the acceptability of health services in terms of comfort with, trust in, or preference for their medical provider. |
| Digital | | | The connectivity that enables synchronous or asynchronous digital communications with formal providers, informal caregivers, peers, and computerized health applications |
| Financial | | | The healthcare system eligibility issues and the cost of utilizing healthcare services |
| **Code 5: Study Design (select one)** | | | |
| Type | | | Definition |
| Observational | | | Secondary data analysis, mixed methods, qualitative methods, modeling |
| Program Evaluation | | | Evaluation of a programmatic initiative designed to improve access |
| Interventional | | | Prospective evaluation of an intervention designed to improve access |
| Note: when a project meets criteria for more than one project design type, final classification is based on the overall goal of the project. (e.g., if a project uses observational methods to inform an intervention that occurred later in the project, it will be classified as an intervention). In cases in which it is difficult to distinguish between project evaluations and interventions, the following decision rules apply: program evaluations are initiatives that were already in the implementation or sustainment stages by the end of the project, whereas interventions are those that were in the pre-implementation phase at the end of the project. | | | |
| **Code 6: OVAC priorities (select primary and secondary themes)** | | | |
| Priority | | | |
| System redesign (PACT integration, clinical delegation, MISSION) | | | |
| Overuse/low-value care/appropriateness | | | |
| Prioritization/urgency/wait list management | | | |
| Virtual care/technology | | | |
| Burnout | | | |
| Workforce satisfaction/retention/expansion | | | |
| Clinical Operations | | | |
| Access measurement | | | |
| Improving patient satisfaction/experience (must have product to address satisfaction) | | | |
|  | IF MORE THAN 1, Rank Top 2 | | |
| **Code 7: Community Care and Virtual Care Focus (select one, both, or note neither)** | | | |
| Type | | Definition | |
| Community Care | | Is there a MISSION or Veterans Choice Act influence or component to the project? | |
| Non-VA Care/Data | | Is there non-VA care or a dataset affiliated with the project unrelated to MISSION or Choice? | |
| Virtual Care | | Does the study involve the use or evaluation of virtual care programs such as secure messaging, mobile apps, telehealth, virtual video connect, etc.)? | |
| **Code 8: Implementation Status (select one)** | | | |
| Implementation not part of objectives | | Project does not address implementation in objectives | |
| Pre-Implementation | | The stage prior to the main implementation - this can include small implementation pilots or demonstration/feasibility projects. | |
| Implementation | | Stage at which implementation has occurred | |
| **Code 9: Non-VA data (Open text)** | | | |
| What non-VA data were considered and/or utilized? [distinguish between (a) non-VA data available through the VA such as community care, CMS data linked to Veterans, fee-basis, and (b) data obtained by an external entity (e.g., Blue Cross, Kaiser, University hospital system, etc.) | | | |
