# Supplementary material for: Engaging Operational Partners Is Critical for Successful Implementation of Research Products: a Coincidence Analysis of Access-Related Projects in the Veterans Affairs Healthcare System
Source: J Gen Intern Med. 2023 Jun 20;38(Suppl 3):923–30. doi: 10.1007/s11606-023-08115-5 (PMC10356702; doi:10.1007/s11606-023-08115-5)
Supplement: Supplementary file 2 — Supplementary file2 (DOCX 32 kb) [file 11606_2023_8115_MOESM2_ESM.docx]

Appendix 2. Access Project Implementation Status Survey

Q1.1 Veteran Access Research Consortium (VARC) “Access CORE”: Survey to Capture Deliverables from Funded Research Projects

The Veteran Access Research Consortium (VARC) is reviewing all access-related VA projects for the purposes of identifying gaps in existing research efforts and opportunities for impact.

The goals of VARC are to accelerate access-related health services research and build a network of researchers (ARC Network) focused on understanding and improving access to care for Veterans.  We have included your [pre-populated: IIR/RRP/etc.] entitled [pre-populated title of project] in VARC’s portfolio review of access-related projects.

**We are interested in hearing about the current status of “deliverables” from your project**—potentially implementable products of your research such as actionable findings or effective tools, models, or interventions. We understand that there often are unexpected and sometimes insurmountable challenges to translating research into practice, so we are interested in hearing both about successes and challenges you have faced in implementing or disseminating your findings.

We appreciate your time and willingness to complete this survey.

As you are completing this survey, please edit any information that is incorrect in the pre-populated fields below.

Q2.2: Study PI (*pre-populated) ______________________________________________________________

Q2.2 Person completing survey, if other than PI (*pre-populated with PI name): ________________________

Q2.3: Job Title ___________________________________________________________________________

Q2.4: Preferred e-mail address (*pre-populated) _________________________________________________

Q2.5: Affiliated VA Office (e.g. COIN, VAMC, etc.) ________________________________________________

Q2.6: For the next set of questions, please think about the following key deliverable that we have identified from your reported results: *[LIST DELIVERABLE HERE]*

*Example deliverables for survey reviewers:*

- *A simulation model to predict increasing patient trends of low-value care*
- *An intervention that uses personalized text messaging to reduce appointment no-shows*

Q2.7: Has the deliverable above been implemented either in whole or in part?

- Completely implemented
- Partially implemented
- Not implemented (includes not currently implemented, but may be planning to implement in the future)

**IF COMPLETELY OR PARTIALLY IMPLEMENTED (Q2.8A – Q2.8G)**

Q2.8A: Is the deliverable demonstrating effectiveness at the sites at which it has been implemented?

- Yes
- No
- Partially
- Not yet examined

Q2.8B: Is the deliverable demonstrating effectiveness at the sites at which it has been implemented?

- Yes
- No
- Partially
- Not yet examined

Q2.8C: In your opinion how important is it that this deliverable be adopted more broadly?

| 1 | 2 | 3 | 4 | 5 | 6 |
| --- | --- | --- | --- | --- | --- |
| Not at  all important |  |  |  |  | Extremely  Important |

| 2.8D: Please rate the following questions about your **deliverable** and its **implementation**: | | | | | | |
| --- | --- | --- | --- | --- | --- | --- |
|  | Strongly disagree | Somewhat disagree | Neither agree nor disagree | Somewhat agree | Strongly agree | n/a |
| Implementation has sufficient resources (e.g., equipment, staff) |  |  |  |  |  |  |
| Implementation has sufficient IT support |  |  |  |  |  |  |
| Implementation was limited by PI's time, expertise, and/or resources |  |  |  |  |  |  |
| The deliverable required substantial modifications prior to broader implementation |  |  |  |  |  |  |
| Implementation creates tension with existing practice (e.g., standards of care, guidelines, policies, systems) |  |  |  |  |  |  |
| Changes in environment have rendered implementation less relevant. |  |  |  |  |  |  |
| Implementation has buy-in from frontline providers and staff |  |  |  |  |  |  |
| Implementation has support and commitment from site leadership |  |  |  |  |  |  |
| Implementation has support and commitment from operational partner |  |  |  |  |  |  |
| Implementation has a champion (leader) at the implementation site |  |  |  |  |  |  |
| Implementation has support and buy-in from key outside community entities |  |  |  |  |  |  |

Q2.8E: Has the deliverable spread to other places outside your original implementation site?

- Yes
- Partially
- No

Q2.8F: Has the deliverable spread to other places inside your original implementation site?

- Yes
- Partially
- No

Q2.8G: Are there other deliverables from this project, that were not part of your original proposal, that you have implemented or intend to implement?

- Yes
- No

**IF NOT IMPLEMENTED (Q2.9A – Q2.9C)**

Q2.9A: In your opinion how important is it that this deliverable be adopted more broadly?

| 1 | 2 | 3 | 4 | 5 | 6 |
| --- | --- | --- | --- | --- | --- |
| Not at  all important |  |  |  |  | Extremely  Important |

| Q2.9B: Thinking about barriers and facilitators, please rate the following statements about the **implementation of your deliverable**: | | | | | | |
| --- | --- | --- | --- | --- | --- | --- |
|  | Strongly disagree | Somewhat disagree | Neither agree nor disagree | Somewhat agree | Strongly agree | n/a |
| Implementation has sufficient resources (e.g., equipment, staff) |  |  |  |  |  |  |
| Implementation has sufficient IT support |  |  |  |  |  |  |
| Implementation was limited by PI's time, expertise, and/or resources |  |  |  |  |  |  |
| The deliverable requires substantial modifications prior to broader implementation |  |  |  |  |  |  |
| Implementation creates tension with existing practice (e.g., standards of care, guidelines, policies, systems) |  |  |  |  |  |  |
| Changes in environment have rendered implementation less relevant. |  |  |  |  |  |  |
| Implementation has buy-in from frontline providers and staff |  |  |  |  |  |  |
| Implementation has support and commitment from site leadership |  |  |  |  |  |  |
| Implementation has support and commitment from operational partner |  |  |  |  |  |  |
| Implementation has a champion (leader) at the implementation site |  |  |  |  |  |  |
| Implementation has support and buy-in from key outside community entities |  |  |  |  |  |  |

Q2.9C: Do you have plans to implement deliverables?

- Yes => When do you anticipate starting the process? (Month/year)
- No

**ALL RESPONSES RESUME REMAINING QUESTIONS**

Q2.10: Which, if any, operational offices have you either contacted or shared your deliverable with?

- None
- Local - please list ________________________________________________
- Regional - please list _______________________________________________
- National - please specify below
- Office of Community Care
- Office of Veterans' Access to Care
- Office of Connected Care
- Office of Specialty Care
- Office of Nursing Service
- Office of Rural Health
- Office of Primary Care
- Office of Mental Health and Suicide Prevention
- Office of Healthy Equity
- Other VA operational partner(s) - please list below _______________________

Q2.11: Would it be okay if we contacted you for additional information?

- Yes
- No

Q2.12: Thank you for your responses.
