# Supplementary material for: Engaging Operational Partners Is Critical for Successful Implementation of Research Products: a Coincidence Analysis of Access-Related Projects in the Veterans Affairs Healthcare System
Source: J Gen Intern Med. 2023 Jun 20;38(Suppl 3):923–30. doi: 10.1007/s11606-023-08115-5 (PMC10356702; doi:10.1007/s11606-023-08115-5)
Supplement: Supplementary file 3 — Supplementary file3 (DOCX 33 kb) [file 11606_2023_8115_MOESM3_ESM.docx]

| **Project Name ^*^** | **Project Goals** | **Study Type^+^** | **Participants** | **Project Deliverables** |
| --- | --- | --- | --- | --- |
| **Development and Validation of a Perceived Access Measure** | Generate a psychometrically sound patient-centered measure of Veterans' perceived access to MH treatments | Observational | Veterans | Perceived Access Inventory (PAI) Tool |
| Leveraging Health Information Technology to improve specialty care access and coordination in Hepatitis C Virus (HCV) | Compare HCV and liver related quality of care among patients enrolled in Specialty Care Access Network–Extension for Community Health Outcomes (SCAN-ECHO) sites to those in control sites; Understand staff perceptions of processes that facilitate collaboration between specialists and generalists and HCV SCAN-ECHO implementation. | Program Evaluation | Veterans, Providers | Project Specialty Care Access Network–Extension for Community Health Outcomes (SCAN-ECHO) Intervention |
| Encouraging Patient-Centered Communication in Clinical Video Telehealth (CVT) Visits | Develop and test a video intervention and develop pamphlets for patients and providers to encourage active and positive communication in CVT medical interactions | Interventional | Providers, Veterans | Video intervention and pamphlets (tool) |
| **VHA-Indian Health Service Collaborations in Rural Health: Home Based Primary Care (HBPC)** | Characterize the organizational contexts and processes of care that account for variation in the HBPC expansion models; Describe the rural HBPC patient population and outcomes of HBPC expansion at patient- and organizational-levels. | Observational | Veterans, Providers, Administrators | Home Based Primary Care (HBPC) collaboration program intervention |
| **Telehealth to Support Antimicrobial Stewardship Implementation at VA Facilities** | Evaluate the use of Videoconference Antimicrobial Stewardship Team (VAST) to improve antimicrobial stewardship at VA facilities with limited access to infectious disease specialists. | Program Evaluation | Providers, Pharmacists, Infection Preventionists, Nurses | Videoconference Antimicrobial Stewardship Team (VAST) Intervention |
| **Improving Access and Outcomes for Rural Veterans with HIV** | Develop, evaluate, and implement innovative delivery models to improve accessibility, quality, and outcomes for rural veterans with HIV | Observational | Veterans, Providers, Administrators | Telehealth Intervention |
| Motivational Coaching to Enhance Mental Health Engagement in Rural Veterans | Adapt, implement, and test motivational coaching intervention to improve mental health (MH) services engagement at community-based outpatient clinics (CBOCs) | Interventional | Veterans | Motivational Coaching Intervention Implementation Toolkit |
| Implementation of Women's Health Patient Aligned Care Teams (WH-PACT) | Develop/test women’s health PACT model. | Program Evaluation | Veterans, Providers, Clinic Staff | Women's Health Patient Aligned Care Teams (WH-PACT) Intervention |
| Controlled Trial of Tele-Support and Education for Women’s Health Care in CBOCs | Evaluate Women's Health Primary Care Providers (DWHP) Support intervention | Program Evaluation | Providers, Other Stakeholders | Quality Measurement Tools |
| Dual Health Systems Users: Strategies to Implement Optimal Care Coordination | Pilot a care coordination intervention | Interventional | Veterans; VA and Non-VA Providers | Bi-directional Information Exchange and Co-management Tool |
| Emergency Care Sensitive Conditions in the VA | Examine access to emergency care and multi-level factors related to emergency care sensitive condition outcomes | Observational | Veterans | Emergency Care Database Tool |
| Implementation of Stigma Reduction Intervention for Primary Care Providers | Test feasibility and impact of an external facilitation strategy to support implementation of SAVE intervention to reduce stigma of mental illness among primary care providers | Interventional | Providers | SAVE  Intervention |
| eHealth Partnered Evaluation Initiative | Implement patient-level secure messaging intervention and evaluate its impact in a 1-year randomized controlled trial; evaluate initial rollout of VHA's automated telehealth text messaging system | Program Evaluation | Veterans, Providers, Clinic Staff | Secure Messaging Intervention |
| **Use of Predictive Modeling to Improve Operating Room Scheduling Efficiency** | Compare impact of predictive modeling system versus traditional scheduling in VA operating rooms | Interventional | Operating Rooms | Predictive Modeling System (PMS) Tool |
| **Telemedicine Outreach for PTSD (TOP) Implementation Project** | Support national deployment of TOP intervention and evaluate clinical effectiveness | Program Evaluation | Providers | Telemedicine Outreach for PTSD (TOP) Intervention |
| **A Multi-Faceted Intervention to Improve Alcohol Dependence (AD) Pharmacotherapy Access** | Increase access to AD pharmacotherapy through intervention targeting Veterans with AD and substance use disorders, primary care mental health integration, and primary care providers; refine intervention and assess costs | Interventional | Veterans, Providers | Alcohol Dependence (AD) Pharmacotherapy Intervention |
| Evaluating Evidence-based Quality Improvement of Comprehensive Women's Health Care in Low-Performing VA Facilities | Evaluate barriers, perceptions and attitudes towards VA Women's Health Services (WHS) /QUERI Partnered Evaluation Initiative | Program Evaluation | Veterans, Providers | WHS Medical Home Intervention |
| Patient-Centered Pain Care Using Artificial Intelligence and Mobile Health Tools | Develop personalized Cognitive Behavioral Therapy pain management service (AI-CBT) intervention or patients with chronic low back pain | Interventional | Veterans | AI-CBT Intervention |
| Relational Agent to Improve alcohol Screening and Treatment in Primary Care: Randomized Control Trial | Improve the primary care management of risky alcohol use among veterans through a computer-based intervention using a “relational agent.” | Interventional | Veterans | Relational Agent Substance use Intervention |
| Patient Aligned Care Team (PACT) for Individuals With Serious Mental Illness | Implement and evaluate a specialized PACT model for individuals with serious mental illness ("SMI-PACT") | Program Evaluation | Veterans | Patient Aligned Care Team for individuals with Serious Mental Illness (SMI-PACT) |
| An Effectiveness Trial of the Triage Algorithm for Patient Aligned Care Team (PACT) Pharmacy Services | Test utility of Triage Algorithm for PACT Pharmacy Services (TAPPS) intervention and examine its effectiveness | Interventional | Pharmacists | Test utility of Triage Algorithm for PACT Pharmacy Services (TAPPS) Intervention |
| The Secure Messaging for Medication Reconciliation Tool (SMMRT) Trial | Evaluating a secure messaging intervention for medication reconciliation | Interventional | Veterans, Pharmacists, Nurses | The Secure Messaging for Medication Reconciliation Tool (SMMRT) Intervention |
| Smart Phone Application for Post-concussion Symptom Reduction | Evaluate utility of an interactive, self-management smartphone application (Traumatic Brain Injury (TBI) Coach) for improving clinical outcomes; improve access to care among Veterans with mild TBI | Interventional | Veterans | TBI Coach Intervention |
| Integrating Care After Exacerbation of Chronic Obstructive Pulmonary Disease (COPD) (InCasE) | Identify vulnerable/high-risk patients, support primary care teams in care of patients recently discharged with COPD exacerbation, integrate proactive collaborative care with specialty care, primary care and pharmacy services, deliver care within the context of existing services and ongoing care, leverage VA IT and virtual care platforms to facilitate improved care delivery, and minimize workload and preserve autonomy for primary care. | Interventional | Veterans, Primary Care Providers | Integrating Care After Exacerbation of Chronic Obstructive Pulmonary Disease (COPD) InCasE Intervention |
| Evaluation of the Initial Deployment of VA's Automated Texting System (Annie) | Examine patient and provider experiences with VA's automated text messaging system | Program Evaluation | Patients, Specialty Providers | Automated Text Messaging Tool (Annie) |
| Abstinence Reinforcement Therapy (ART) for Rural Veteran Smokers | Evaluate effectiveness of intervention that combined evidenced based treatment for smoking cessation with smartphone-based tool on smoking rates compared to a contact control intervention in a randomized controlled trial among Veteran smokers | Interventional | Veterans | Smoking cessation Intervention |
| Telemedicine management of veterans with PTSD and chronic insomnia | Determine if Veterans with PTSD and chronic insomnia receiving Cognitive Behavioral Therapy for insomnia (CBT-I) via telemedicine have improvement in insomnia severity compared to Veterans receiving in-person CBT-I | Interventional | Veterans | Telemedicine Cognitive Behavioral Therapy for insomnia (CBT-I) Intervention |
| **Web and Shared Decision Making for Reserve/National Guard Women's Post-Traumatic Stress Disorder (PTSD) Care** | Evaluation of Veterans Health Administration (VHA) Mental Health services and evidence-based practice for Women Veterans with PTSD | Interventional | Operation Enduring Freedom / Operation Enduring Freedom / Operation Iraqi Freedom / Operation New Dawn / Reserve National Guard female war Veterans | Provider Engagement Tool |
| Comparison of E-Health vs. In-Person Delivered Family Psychoeducation Treatment | Non-inferiority randomized trial of Schizophrenia On-line Access to Resources (SOAR) intervention | Program Evaluation | Veterans | Non-inferiority randomized trial of Schizophrenia On-line Access to Resources (SOAR)  Intervention |
| Optimizing eHealth Applications for Multimorbid Patients | Understand self-management and health care navigation challenges faced by Veterans with multiple chronic conditions; identify eHealth technology and health care delivery interventions | Observational | Veterans | Multimorbidity conceptual framework  (Tool) |
| Evaluation of Web-Based CBT for Women Veterans with PTSD | Develop low cost, accessible interventions for women Veterans with PTSD. | Interventional | Veterans | Web-based  Intervention |
| Family Tele-Mental Health Intervention for Veterans with Dementia | Identify and address caregiver interpersonal challenges using telehealth technology. | Interventional | Veterans, Veteran Caregivers, Providers | Video Based Intervention |

^*^Projects classified as access specific are bolded. ^+^ Definitions of study type are as follows: Observational - Secondary data analysis, mixed methods, qualitative methods, modeling; Program Evaluation - Evaluation of a programmatic initiative designed to improve access; Interventional - Prospective evaluation of an intervention designed to improve access; N/A means that there were not participants necessary for project.
